# Therapeutic Effects of Lithospermate B Complexed with Mg^2+^ or Zn^2+^ on Metabolic Syndrome Induced in Rats Fed with High-Fat Diet

**DOI:** 10.3390/molecules25040983

**Published:** 2020-02-22

**Authors:** Sheng-Kuo Hsieh, Nan-Hei Lin, Ying-Jie Chen, Maw-Rong Lee, Wen-Ying Chen, Jason T.C. Tzen

**Affiliations:** 1 Graduate Institute of Biotechnology, National Chung-Hsing University, Taichung 402, Taiwan; vincentqaz123@gmail.com (S.-K.H.); pp.club@msa.hinet.net (Y.-J.C.); 2 Department of Biotechnology and Pharmaceutical Technology, Yuanpei University of Medical Technology, Hsinchu 300, Taiwan; CMNHEI@mohw.gov.tw; 3 Department of Chemistry, National Chung-Hsing University, Taichung 402, Taiwan; mrlee@dragon.nchu.edu.tw; 4 Department of Veterinary Medicine, National Chung-Hsing University, Taichung 402, Taiwan

**Keywords:** high-fat diet, insulin resistance, lithospermate B, metabolic syndrome, zinc

## Abstract

Excessive food consumption and insufficient exercise lead to the prevalence of metabolic syndrome in modern life, which consequently increases the risk of many chronic diseases. Magnesium lithospermate B (MLB) from Danshen has been demonstrated to improve metabolic changes in high-fat diet–fed rats with metabolic syndrome. In this study, Mg^2+^ in MLB was successfully replaced with Zn^2+^ to form zinc lithospermate B (ZLB) complex. MLB (10 mg/kg /day) and ZLB of various concentrations (1, 2.5, 5, and 10 mg/kg/day) were prepared and examined for their therapeutic effects on metabolic syndrome induced in rats fed with a high-fat diet. The results showed that both MLB and ZLB were able to recover or alleviate the abnormal physiological states of high-fat diet–fed rats including weight gain, epididymal fat accumulation, fatty liver, retarded blood lipid and glucose metabolism putatively caused by insulin resistance, and elevated levels of proinflammatory cytokine, leptin, and oxidative stress. In an overall view of the animal study, the effectiveness of ZLB supplementation seemed to be better than that of MLB supplementation for the recovery of high-fat-fed rats from metabolic syndrome.

## 1. Introduction

Metabolic syndrome is an emerging health problem worldwide. It is clinically defined by a group of metabolic risk determinants, including central obesity, hypertension, dyslipidemia, glucose intolerance, and insulin resistance [1,2,3,4]. Among the risk determinants, insulin resistance represents a central player in the development of metabolic syndrome [5]. Thus, an improvement of insulin sensitivity in vulnerable subjects seems to be an adequate therapeutic approach for the treatment of metabolic syndrome.

Clinical and experimental findings indicate that changes of dietary habit and lifestyle along with supplement with nutraceuticals have benefits against metabolic syndrome [2]. Increasing evidence shows that many herbal extracts and natural compounds have been implicated in the treatment of metabolic syndrome [6,7,8,9,10,11,12,13]. Particularly, *Salvia miltiorrhiza* (Danshen), a traditional Chinese medicine used for more than 2000 years, has been frequently prescribed to patients with cardiovascular and metabolic diseases, including diabetes [14,15,16,17,18,19,20].

Lithospermate B (LSB) is the most abundant polyphenolic compound in the soluble extracts of Danshen [18]. Its inhibitory effect on Na^+^,K^+^-ATPase is suggested to be possibly responsible for the cardiovascular effects of Danshen [21]. Natural occurring LSB, termed MLB, is a salt formed as a result of the incorporation of Mg^2+^ with the four oxygen atoms of the carboxyl groups originated from the four caffeic acid fragments [21]. In vitro, the binding metal, Mg^2+^ on LSB could be replaced with transition metals, such as Zn^2+^, Cr^3+^, Mn^2+^, Co^2+^, and Ni^2+^, and these transition metal–LSB complexes were found to be more effective in inhibiting Na^+^/K^+^-ATPase than LSB and MLB [22].

Previously, we reported that MLB was able to improve metabolic changes in high-fat diet–fed rats with metabolic syndrome [23]. In this study, we aimed to develop a metal complex with LSB for the potential treatment of metabolic syndrome better than MLB. In light of the safety concern for oral administration with transition metals, zinc ion, an important trace element for human health, was used to complex with LSB. To test its relative efficacy, Zn–LSB complexes (ZLB) of various concentrations as well as MLB were prepared and subjected to the evaluation of therapeutic effects on metabolic syndrome in a rat model induced by high-fat diet (HFD).

## 2. Results

### 2.1. Preparation and Identification of LSB Complexed with Mg^2+^ or Zn^2+^

**Figure 1:**
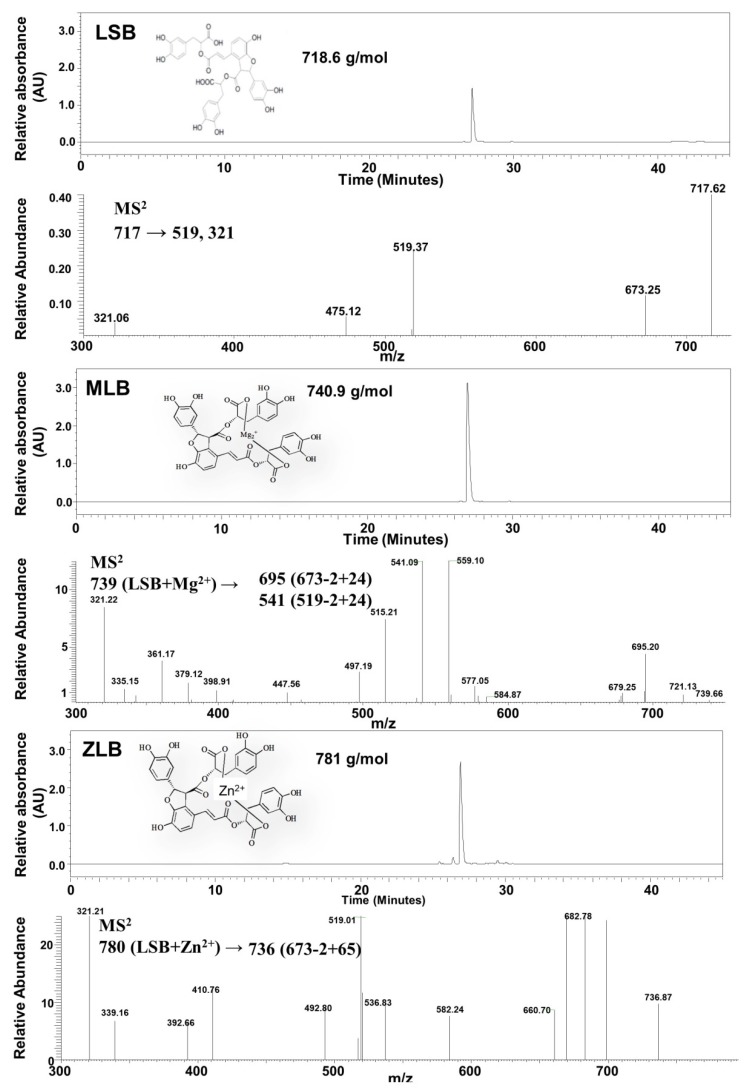
Identification of magnesium lithospermate B (MLB) and zinc lithospermate B (ZLB) by HPLC and LC−MS. Lithospermate B (LSB), MLB, and ZLB were detected by HPLC spectrum at 280 nm and confirmed by mass spectrometric analyses on the basis of fragmentation patterns of LSB published previously [24,25].

Mg-LSB (MLB) and Zn-LSB (ZLB) were prepared by incorporating LSB with Mg^2+^ and Zn^2+^, respectively. To confirm the success of incorporation, LSB, MLB, and ZLB were subjected to HPLC/UV and MS analyses (Figure 1). LSB and MLB were identified by comparing with the HPLC/UV profiles and MS data published previously [24,25]. ZLB was identified by comparing its fragmentation pattern, *m/z* 780 → 736 (673 − 2 + 65) with the MLB fragmentation pattern, *m/z* 739 → 695 (673 − 2 + 24). MLB and ZLB of high purity as detected in the HPLC/UV chromatograms were used in the following animal study.

### 2.2. Effects of MLB and ZLB on Appetite and Weight Gain of Rats

**Figure 2:**
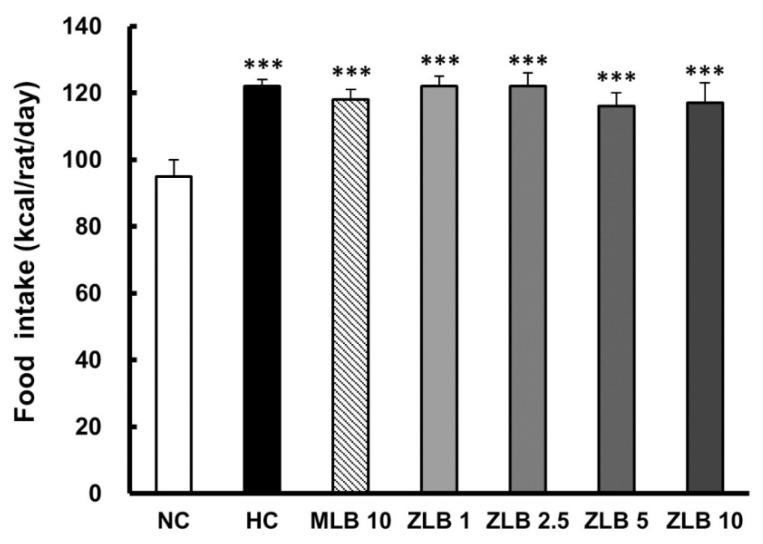
Effects of MLB and ZLB on appetite and weight gain. Food intake was recorded for a month. NC, normal diet control; HC, high-fat-diet (HFD) control; MLB10, HFD with 10 mg/kg/day of MLB; ZLB1, ZLB2.5, ZLB5, and ZLB10, HFD with 1, 2.5, 5, and 10 mg/kg/day of ZLB. Values are expressed as mean ± SD (n = 6 per group). ***P < 0.001 vs. NC group.

In the duration of 30 days, the food intake of rats fed with an HFD was found to be higher than that of rats fed with normal diet regardless of whether MLB or ZLB was supplemented daily (Figure 2). Apparently, supplementation of MLB or ZLB to rats did not upset their appetite. Correspondingly, the body weights of rats fed with an HFD were detected to increase steadily in comparison with those of rats fed with normal diet (Table 1). The increase of body weight in rats fed with an HFD was substantially reduced when MLB or ZLB was supplemented. The reduction in body weight gain by ZLB supplementation was found to be dose dependent in the concentration ranging from 1 to 10 mg/kg BW/day.

### 2.3. Effects of MLB and ZLB on Epididymal Fat and Liver Injury

**Figure 3:**
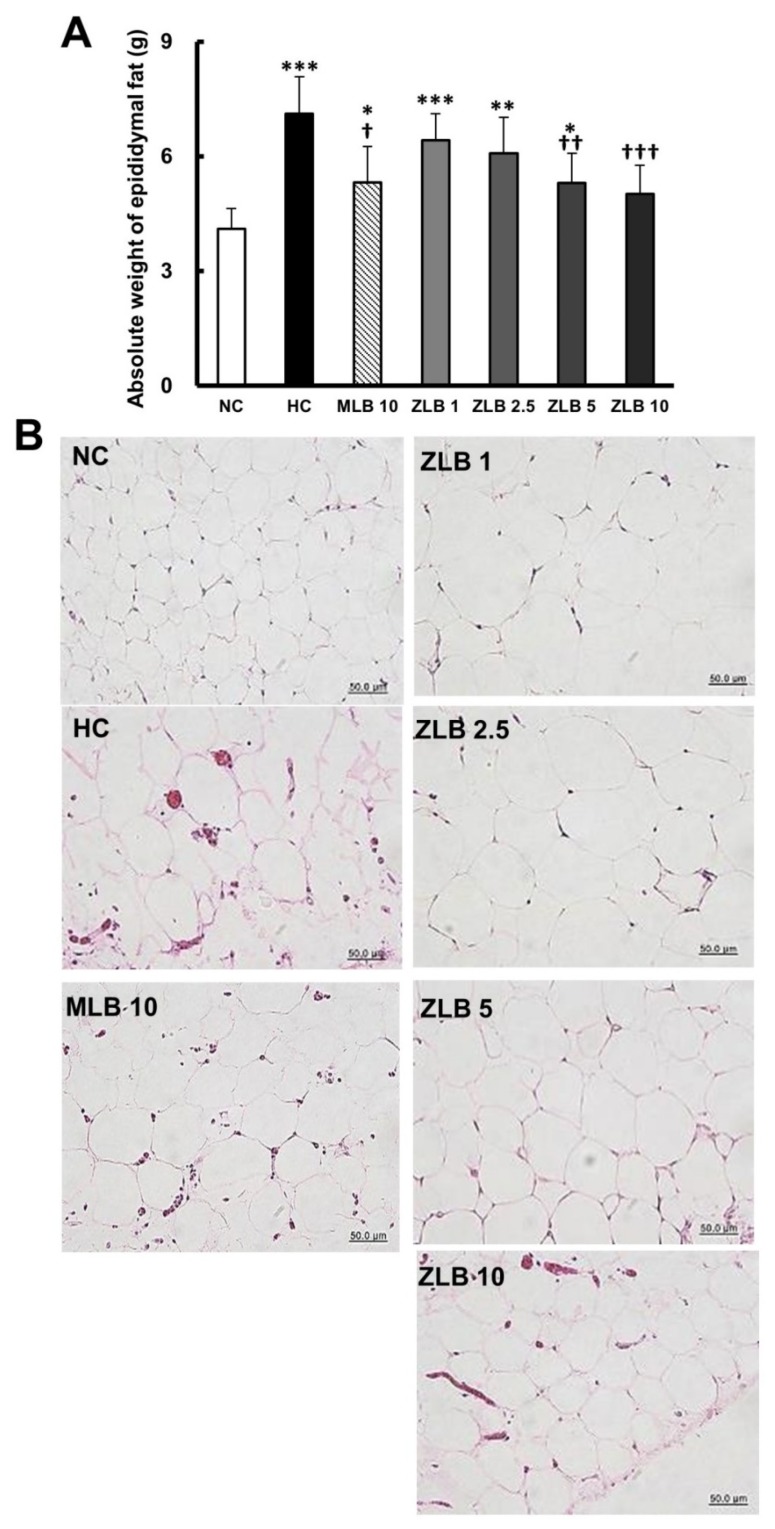
Effects of MLB and ZLB on epididymal fat. The rats were sacrificed at the end of the experiment (a month). Weight of the epididymal fat was measured (**A**), and representative photomicrographs of epididymal fat tissues are shown at a magnification of ×400 (**B**). NC, normal diet control; HC, high-fat-diet (HFD) control; MLB10, HFD with 10 mg/kg/day of MLB; ZLB1, ZLB2.5, ZLB5, and ZLB10, HFD with 1, 2.5, 5, and 10 mg/kg/day of ZLB. Values are expressed as mean ± SD (n = 6 per group). *P < 0.05, **P < 0.01, and ***P < 0.001 vs. NC group; ^†^*P* < 0.05, ^††^*P* < 0.01, and ^†††^*P* < 0.001 vs. HC group.

The weight of epididymal fat of rats fed with an HFD was found to be significantly higher than that of rats fed with normal diet (Figure 3A). The upsurge of epididymal fat in rats fed with an HFD was substantially abridged when MLB or ZLB was supplemented, and the abridgement of epididymal fat by ZLB supplementation was observed to be dose dependent. As expected, the adipocytes in the epididymal fat of rats fed with an HFD were enlarged in comparison with those of rats fed with a normal diet, and the enlarged adipocytes became smaller by the supplementation of MLB or ZLB dose dependently (Figure 3B).

**Figure 4:**
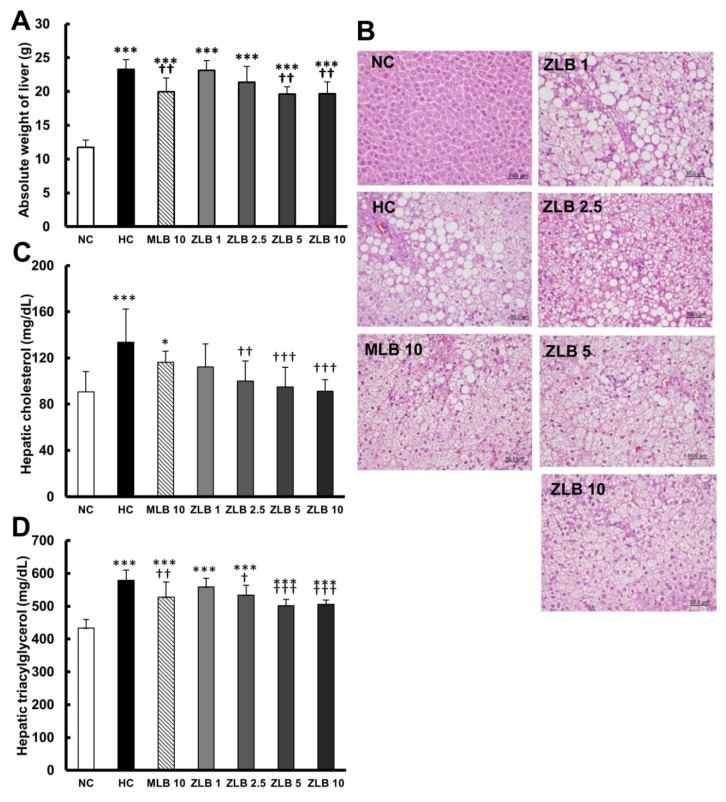
Effects of MLB and ZLB on lipid deposition in the liver. Weight of the liver was measured (**A**), and representative photomicrographs of liver tissues are shown at a magnification of ×400 (**B**). The levels of cholesterol (**C**) and triacylglycerol (**D**) in the liver tissues were measured. NC, normal diet control; HC, high-fat-diet (HFD) control; MLB10, HFD with 10 mg/kg/day of MLB; ZLB1, ZLB2.5, ZLB5, and ZLB10, HFD with 1, 2.5, 5, and 10 mg/kg/day of ZLB. Values are expressed as mean ± SD (n = 6 per group). *P < 0.05, ***P < 0.001 vs. NC group; ^†^*P* < 0.05, ^††^*P* < 0.01, and ^†††^*P* < 0.001 vs. HC group.

The weight of the liver of rats fed with an HFD was found to be significantly higher than that of rats fed with normal diet; however, the upsurge in liver weight could be partially reduced when MLB or ZLB was supplemented (Figure 4A). Morphological examination of the liver showed that substantial fat was deposed in the hepatocytes of rats fed with an HFD, and the abnormal hepatocytes became larger and their color turned from red to white (Figure 4B). The size and color of hepatocytes of rats fed with an HFD could be substantially recovered by the supplementation of MLB or ZLB. The levels of hepatic cholesterol (Figure 4C) and triacylglycerol (Figure 4D) of rats fed with an HFD were significantly elevated when compared with those of rats fed with normal diet, and the elevated levels were dose-dependently diminished by the supplementation of ZLB. Meanwhile, the levels of aspartate transaminase (AST) and alanine transaminase (ALT) in plasma of HFD-fed rats were detected to be higher than those of normal rats, and MLB or ZLB supplementation seemed to significantly reduce these two levels of enzymatic activities back to the control levels of normal rats (Table 2).

### 2.4. Effects on Kidney Indices and Lipid Metabolism

To understand the safety of ZLB, two kidney indices, blood urea nitrogen (BUN) and creatinine were detected; and the data showed that these two indices were not significantly affected in rat blood regardless of the supplementation of MLB or ZLB (Table 2). The levels of total cholesterol, triacylglycerol, and non-HDL-cholesterol in the plasma of HFD-fed rats were detected to be significantly raised in comparison with those of normal rats. The raised levels of these three lipid contents were mostly adjusted back to normal levels by the supplementation of MLB or ZLB. In contrast, the plasma HDL level of HFD-fed rats was obviously lowered compared to that of normal rats, and the low HDL level could be rescued back to the normal level by the supplementation of MLB or ZLB.

### 2.5. Effects on Glucose Metabolism

**Figure 5:**
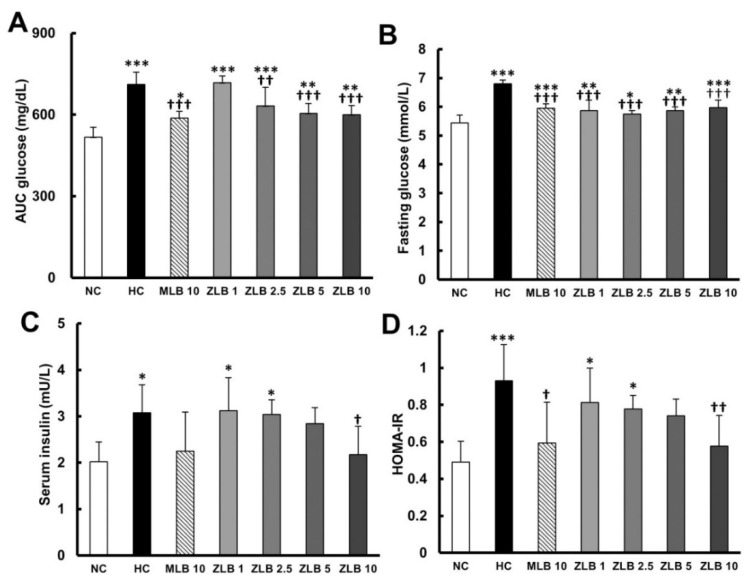
Effects of MLB and ZLB on glucose metabolism. The glucose tolerance test was carried out three weeks after administration of MLB or ZLB. The levels of blood glucose were measured every 30 min for 2 h, and the area under the curve (AUC) of the blood glucose curve is depicted (**A**). The levels of fasting blood glucose (**B**) and insulin (**C**) were measured at the end of the experiment (4 weeks). The homeostatic model assessment for insulin resistance (HOMA-IR) data were calculated and depicted (**D**). NC, normal diet control; HC, high-fat-diet (HFD) control; MLB10, HFD with 10 mg/kg/day of MLB; ZLB1, ZLB2.5, ZLB5, and ZLB10, HFD with 1, 2.5, 5, and 10 mg/kg/day of ZLB. Values are expressed as mean ± SD (n = 6 per group). *P < 0.05, **P < 0.01, and ***P < 0.001 vs. NC group; ^†^*P* < 0.05, ^††^*P* < 0.01, and ^†††^*P* < 0.001 vs. HC group.

To examine glucose tolerance in rats, glycemia stabilization was detected at the end of the third week. A relatively slow rate of glycemia stabilization was observed in rats fed with an HFD in comparison with rats fed with normal diet (Table 3 and Figure 5A). The rate of glycemia stabilization in HFD-fed rats could be dose-dependently enhanced by ZLB supplementation. Fasting glucose and serum insulin were detected at the end of the fourth week. The results showed that both fasting glucose and serum insulin in HFD-fed rats were found to be higher than those in normal rats, and the higher levels of fasting glucose and serum insulin could be reduced by the supplementation of MLB or ZLB (Figure 5B,C). Furthermore, the level of homeostatic model assessment for insulin resistance (HOMA-IR) in HFD-fed rats was significantly higher than that in normal rats (Figure 5D), indicating HFD-fed rats were prediabetic and insulin resistance. However, the high level of HOMA-IR was significantly decreased by the supplementation of MLB or ZLB.

### 2.6. Effects on Intracellular Signaling in Lipid and Glucose Metabolism

**Figure 6:**
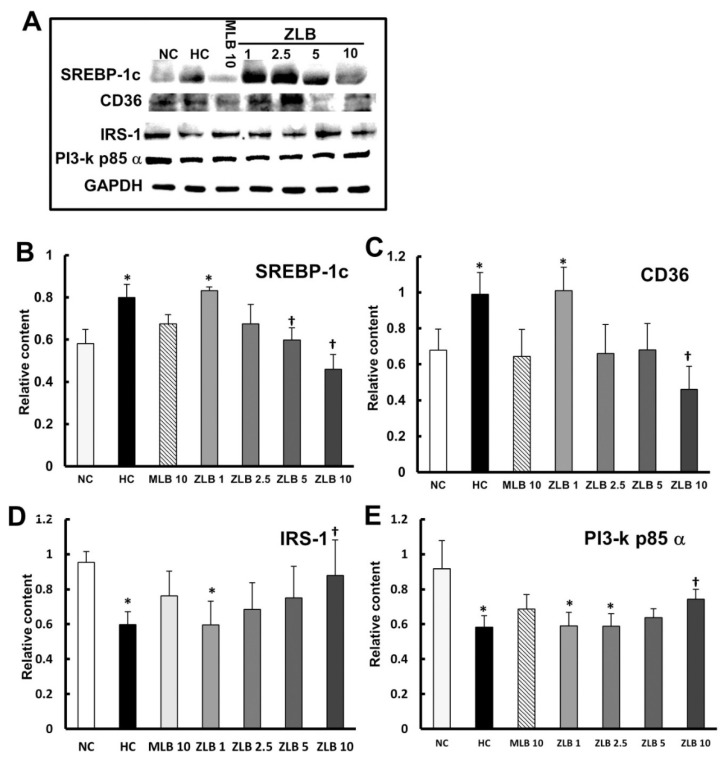
Effects of MLB and ZLB on intracellular signaling in metabolism. Marker proteins for liver lipid and glucose metabolism were detected by Western blotting (**A**). GAPDH was used as an internal standard. The blots of SREBP1c (**B**), CD36 (**C**), IRS-1 (**D**), and PI3-kp85α (**E**) are calculated and shown in the bar chart. NC, normal diet control; HC, high-fat-diet (HFD) control; MLB10, HFD with 10 mg/kg/day of MLB; ZLB1, ZLB2.5, ZLB5, and ZLB10, HFD with 1, 2.5, 5, and 10 mg/kg/day of ZLB. Values are expressed as mean ± SD (n = 6 per group). *P < 0.05 vs. NC group and ^†^*P* < 0.05 vs. HC group.

To examine the intracellular signal transduction of lipid and glucose metabolism affected by insulin, marker proteins in liver tissues were detected by Western blotting (Figure 6A). The results showed that protein expression levels of SREBP1c and CD36 (regulating fatty acid lipid metabolism) were enhanced in liver tissues of HFD-fed rats in comparison with those of normal rats, and the enhanced levels could be diminished by the supplementation of MLB or ZLB dose dependently (Figure 6B,C). The protein expression levels of IRS-1 and PI3-k p85α transmitting signals for insulin) declined in liver tissues of HFD-fed rats in comparison with those of normal rats, and the declined levels could be rescued by the supplementation of MLB or ZLB dose dependently (Figure 6D,E).

### 2.7. Effects on Proinflammatory and Oxidative Stress

Chronic inflammation is a crucial index in metabolic syndromes. As expected, the plasma levels of tumor necrosis factor α (TNFα) and leptin that were elevated in HFD-fed rats compared with those of normal rats (Table 2) in accord with a previous study showing that obesity and insulin resistance would increase the plasma level of proinflammatory cytokines and adipokines [26]. Similarly, the elevated levels of TNFα and leptin in HFD-fed rats were significantly reduced by the supplementation of MLB or ZLB.

**Figure 7:**
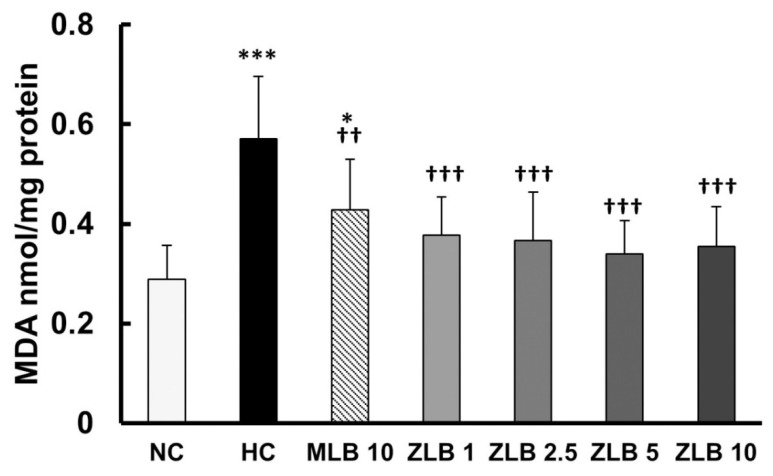
Effects of MLB and ZLB on oxidation stress. Malondialdehyde (MDA), a byproduct of lipid peroxidation, was measured by a thiobarbituric acid reactive substances (TBARS) assay as an indicator of oxidative stress. NC, normal diet control; HC, high-fat-diet (HFD) control; MLB10, HFD with 10 mg/kg/day of MLB; ZLB1, ZLB2.5, ZLB5, and ZLB10, HFD with 1, 2.5, 5, and 10 mg/kg/day of ZLB. Values are expressed as mean ± SD (n = 6 per group). *P < 0.05 and ***P < 0.001 vs. NC group; ^††^*P* < 0.01 and ^†††^*P* < 0.001 vs. HC group.

Thiobarbituric acid reactive substances (TBARS), byproducts of lipid peroxidation, are indicators of oxidative stress. In this study, malondialdehyde (MDA), a byproduct of lipid peroxidation, was measured as an indicator of oxidative stress. The results showed that the level of oxidative stress in rats fed with an HFD for a month was significantly higher than that in rats fed with normal diet, and the higher level of oxidative stress was alleviated by the supplementation of MLB or ZLB (Figure 7).

### 2.8. Zinc Concentration

**Figure 8:**
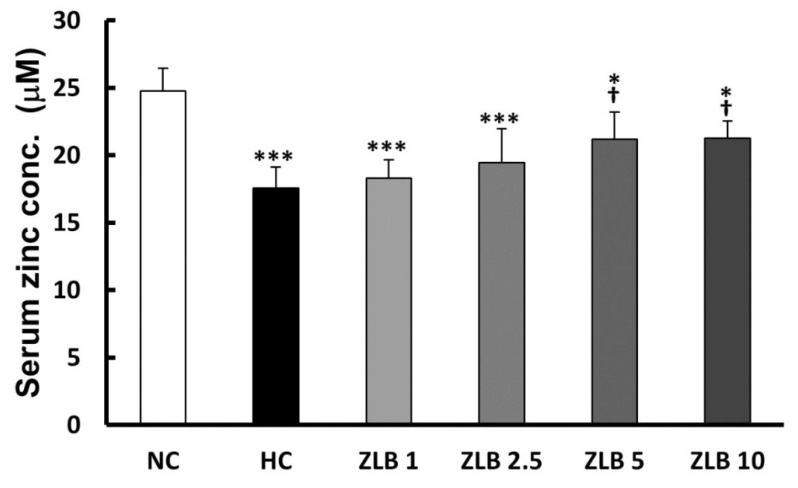
Serum Zn^2+^ concentration after ZLB administration. The serum Zn^2+^ concentration was measured by a zinc colorimetric assay kit. NC, normal diet control; HC, high-fat-diet (HFD) control; ZLB1, ZLB2.5, ZLB5, and ZLB10, HFD with 1, 2.5, 5, and 10 mg/kg/day of ZLB. Values are expressed as mean ± SD (n = 6 per group). *P < 0.05 and ***P < 0.001 vs. NC group; ^†^*P* < 0.05 vs. HC group.

The rat serum samples were subjected to the analysis of zinc concentration. The results showed that Zn^2+^ level decreased in the HFD group compared with the control group. After ZLB administration, the Zn^2+^ level was elevated dose dependently, particularly, ZLB 5 and ZLB 10 groups showed significant elevation in serum Zn^2+^ concentration (Figure 8).

## 3. Discussion

In this study, obesity, hyperglycemia, hyperlipidemia, insulin resistance, and hepatic steatosis were induced in rats by feeding an HFD for a month. These abnormal physiological states were significantly relieved by the supplementation of MLB or ZLB dose dependently. In an overall view of the animal study, the effectiveness of ZLB supplementation seemed to be better than that of MLB supplementation for the recovery from metabolic syndrome. The pharmacological effects of MLB or ZLB were presumably attributable to the enhancement of lipid and glucose metabolism as well as the improvement of insulin sensitivity. Insulin resistance is regarded as a primary causative factor and pathophysiological basis of metabolic syndrome; and it has been demonstrated that increasing insulin sensitivity could significantly alleviate those diseases associated with metabolic syndromes [27]. The findings suggest that ZLB as well as MLB may be an alternative nutraceutical with beneficial effects against metabolic syndrome. Of course, more investigation should be executed before their clinical applications.

Metabolic syndrome is associated with a cluster of pathological conditions, such as low-grade chronic inflammation and oxidation states [28,29]. Polyphenolic compounds, the most widely distributed secondary metabolites in plants, show biological activities in the alleviation of the chronic inflammation and oxidation states [30]. Curcumin and resveratrol are two well-known polyphenolic compounds displaying anti-oxidant and anti-inflammatory activity resulting in the improvement of metabolic syndrome [31,32]. LSB is a derivative of a caffeic acid tetramer, showing anti-oxidative and free-radical scavenging activities [33,34,35,36,37,38,39]. Thus, the LSB backbone of the ZLB and MLB is assumed to be the central player for protecting from metabolic syndrome. Because of the low bioavailability of LSB [24,39,40,41], its effect alone against metabolic syndrome was not investigated in this study. Alternatively, the beneficial effects of ZLB and MLB may come from the increase of their bioavailability. Since their dynamic bioavailability was not measured, this assumption requires further investigation.

There were interesting findings in the current study. It appeared that ZLB and MLB shared comparable protection against metabolic syndrome. Other than the LSB backbone, zinc and magnesium are the candidates for the discrepancy between ZLB and MLB. Zinc is known in balancing the oxidant/anti-oxidant system and also in improving insulin resistance [42]. Furthermore, zinc and magnesium have direct and indirect effects on the secretion and signal transduction of insulin and in the development of insulin resistance [43,44]. Possibly, the complexed zinc or magnesium ion might be released from ZLB or MLB after intravenous injection into the rodents. Therefore, the protective effects of ZLB or MLB may be secondary to the dissociation reaction of salt–LSB complexes. However, this hypothesis was not addressed in the current study.

Danshen is an essential and reliable herb or food for health promotion [17]. Other than polysaccharides and tanshinones, using an HFD rat model, our findings further provide evidence showing that the polyphenolic compounds, ZLB and MLB, represent alternative ingredients of Danshen in the protection against metabolic syndrome. Although there is still a lack of a fully mechanistic exploration, current findings suggest that the LSB backbone and zinc/magnesium ion may be mediators of the protection. Moreover, the bioavailability of the salt form of LSB complexes is another concern. Despite the encouraging findings of ZLB and MLB against metabolic syndrome, the detailed underlying mechanisms warrant further investigation.

## 4. Materials and Methods

### 4.1. Preparation of LSB Complexed with Mg^2+^ and Zn^2+^

LSB was purchased from KO DA Pharmaceutical Co., Ltd. (Taoyuan, Taiwan). Mg-LSB (MLB) or Zn-LSB (ZLB) complex was prepared by incorporating LSB with Mg^2+^ or Zn^2+^ according to the protocol developed previously [22]. Briefly, LSB was dissolved in 1 mL of H_2_O to a final concentration of 10 mM, and then mixed with 10 mM of Mg(OH)_2_ or Zn(OH)_2_ to form MLB or ZLB. MLB and ZLB were dried by lyophilization and stored at 4 °C. Prior to utilization in the following studies, MLB and ZLB were dissolved in H_2_O to desired concentrations.

### 4.2. HPLC/UV and LC−MS^n^ Analyses of LSB, MLB, and ZLB

Solutions of LSB, MLB, and ZLB were filtered through a 0.22 μm polyvinylidene difluoride (PVDF) membrane filter (Pall Corporation, Glen Cove, NY, USA) and subjected to HPLC analysis. The HPLC system coupled to a model 600E photodiode array detector (Waters Corporation, Milford, MA, USA) was performed using a Syncronis C18 column (4.6 × 250 mm inner diameter, 5 µm, Thermo Scientific, Waltham, MA, USA) with an eluting gradient as follows: 5% acetonitrile for 0–5 min; linear gradient from 5% to 70% acetonitrile for 5–35 min; 70% acetonitrile for 35–45 min. The acetonitrile solution used in the eluting gradient contained 0.5% acetic acid. The ultraviolet (UV) absorbance was detected at 280 nm. Mass spectrometric analysis was performed on a linear trap quadrupole tandem mass spectrometer (Thermo Electron, San Jose, CA, USA) equipped with an electrospray ionization interface and connected to a Surveyor LC system (Thermo Electron, Waltham, MA, USA) with a 5 μL sample loop. The analytes were separated under the same condition used for HPLC analysis. Negative ESI mode was firstly scanned ranging from *m*/*z* 150 to 1500. The other scans were set as the data-dependent MS*^n^* scan using the high-purity helium (>99.99%) as the collision gas and the relative collision energy of 30%.

### 4.3. Animal and Drug Administration

Male Sprague–Dawley rats of weight 150–170 g were obtained from BioLASCO, Taiwan Co., Ltd. (Taipei, Taiwan) and adapted for 1 week before experiments. Animals were kept in a standard, controlled room of 23 ± 2 °C, 60% ± 10% humidity, and 12 h light/dark cycle and fed with a standard chow diet (calories provided by 28.5% protein, 13.5% fat, and 58% carbohydrate, 5001 Rodent LabDiet, St. Louis, MO, USA) or a high-fat diet (HFD, calories provided by 16% protein, 39.4% fat, and 44.6% carbohydrate, high-fat 5S8X Rodent TestDiet, St. Louis, MO, USA) for a month. Animals were randomly divided into seven groups (n = 6 per group): (1) NC group (fed with standard chow diet); (2) HC group (fed with an HFD); (3) MLB 10 group (fed with an HFD and administered with 10 mg/kg BW/day of MLB; (4) ZLB 1 group (fed with an HFD and administered with 1 mg/kg BW/day of ZLB); (5) ZLB 2.5 group (fed with an HFD and administered with 2.5 mg/kg BW/day of ZLB); (6) ZLB 5 group (fed with an HFD and administered with 5 mg/kg BW/day of ZLB); (7) ZLB 10 group (fed with an HFD and administered with 10 mg/kg BW/day of ZLB). The animal experiments were approved by the Institutional Animal Care and Use Committee of the National Chung−Hsing University with the IACUC Approval Number: 103-31.

### 4.4. Intraperitoneal Glucose Tolerance Test and Homeostatic Model Assessment Index

Intraperitoneal glucose tolerance test (IPGTT) was carried out three weeks after MLB or ZLB administration. After fasting for 12 h, rats were treated with glucose solution 2 g/kg BW via intraperitoneal injection. The glycemia was examined by using the ACCU-CHEK^®^ active glucose meter with test strips (Roche, Mannheim, Germany) at 0, 30, 60, 90, and 120 min. The area under the curve (AUC) of the glycemia level was calculated for glucose tolerance. Insulin resistance was observed from fasting glucose and insulin level by calculating via the homeostatic model assessment for insulin resistance (HOMA-IR) index formula. HOMA-IR = fasting insulin (FPI: mU/L) × fasting glucose (FPG: mmol/L)/22.5 [45].

### 4.5. Liver Lipid Extraction and Measurement

Liver lipids were extracted by following the protocol developed previously [46]. Liver tissues were homogenized in 0.15 M NaCl (Sigma-Aldrich, St. Louis, MO, USA), and the tissue pulps were mixed with chloroform/methanol (2:1, *v*/*v*) (Sigma-Aldrich). After centrifugation at 400× *g* for 5 min, the supernatants containing liver lipids were collected. Immediately, the supernatants were measured with an enzymatic colorimetric assay (Human Gesellschaft für Biochemica und Diagnostica mbH, Wiesbaden, Germany) to examine the levels of hepatic cholesterol and triacylglycerol.

### 4.6. Analysis of Blood Biochemistry

After MLB or ZLB administration for four weeks, the rats were fasted overnight and sacrificed. Whole-blood samples were collected to analyze blood biochemistry. The levels of aspartate transaminase (AST), alanine transaminase (ALT), blood urine nitrogen (BUN), creatinine, total cholesterol (TC), triacylglycerol (TAG), high-density lipoprotein cholesterol (HDL-C), and non-HDL-C were analyzed by automated standardized procedures (Hitachi 717). The levels of insulin (Mercodia AB, Uppsala, Sweden), tumor necrosis factor-α (TNF-α), and leptin (Quantikine R&D Systems, Minneapolis, MN, USA) were measured by rat enzyme-linked immunosorbent assay (ELISA) kits.

### 4.7. Histological Examination

The epididymal fat and liver were removed from rats and collected for further examination. These tissue specimens were fixed in 10% formalin solution and embedded in paraffin. Then the tissue specimens were stained with hematoxylin and eosin (H&E). Histological images were taken by a digital camera (Cannon EOS 600D, Tokyo, Japan) under a light microscope (Olympus, BX43, Tokyo, Japan).

### 4.8. Tissue Preparation and Western Blot Analysis

After the rats were scarified, the gastrocnemius muscle and liver were quickly frozen in liquid nitrogen, and stored at −80 °C. The tissues were homogenized with the T-PER™ tissue protein extraction buffer (Pierce Biotechnology, Thermo-Fisher, Rockford, IL, USA) containing 1% protease inhibitor cocktail and 1% phosphatase inhibitor cocktail (Calbiochem, Merck Millipore, Darmstadt, Germany). The protein samples in the supernatants were collected after centrifugation at 15,000× *g* at 4 °C for 10 min, and analyzed by Western blotting with primary antibodies detecting IRS-1 (1:1000, GeneTex, San Antonio, TX, USA), PI3-k p85 α (1:1000; Cell Signaling Technology, Beverly, MA, USA), SREBP1c, CD36 (1:1000; Novus Biologicals, Littleton, CO, USA), and Glyceraldehyde 3-phosphate dehydrogenase (GAPDH, 1:20,000; Merck Millipore), and then detected with secondary antibodies (anti-rabbit or anti-mouse IgG HRP-conjugated). The blots were quantified by chemiluminescence with MiniChemi I system (Beijing Sage Creation Science, Beijing, China), and then normalizing with GAPDH. The relative protein intensity was expressed as folds of the content in the NC group.

### 4.9. Thiobarbituric Acid Reactive Substances Assay

A part of the frozen liver tissue was homogenized in PBS at a concentration of 50 mg/mL, and the supernatant was measured by the thiobarbituric acid reactive substances (TBARS) assay kit (BioAssay System, Hayward, CA, USA) to detect the level of malondialdehyde (MDA) generated from lipid hydroperoxide as an indicator of lipid peroxidation. To normalize the MDA level, total-protein contents in the samples were measured by a protein assay reagent (Bio-Rad, Hercules, CA, USA).

### 4.10. Zinc Concentraction Assay

The Zn^2+^ level of the serum was assayed with a zinc colorimetric assay kit (BioVision, Milpitas, CA, USA). The serum samples were deproteinized by 7% trichloroacetic acid (TCA) solution with a 1:1 ratio (serum: 7% TCA). The supernatants were mixed with zinc-detection reagents and subsequently detected at 560 nm.

### 4.11. Statistical Analysis

The data were presented as mean values ± standard deviation (SD). The differences were analyzed by one-way analysis of variance (ANOVA) followed by Duncan’s post-hoc testing. Statistical calculations were performed by SigmaStat (version 3.5). A level of *p* < 0.05 was considered to be statistically significant.

## 5. Conclusions

Previously, MLB was demonstrated to improve metabolic changes in high-fat diet–fed rats with metabolic syndrome. In this study, we replaced Mg^2+^ in MLB with Zn^2+^ to form the ZLB complex. The therapeutic effects of ZLB supplementation were shown to be better than those of MLB supplementation for the recovery of high-fat-fed rats from metabolic syndrome. It seems that ZLB possesses a great potential to be developed as a beneficial supplement for patients suffering from metabolic syndrome.

## Figures and Tables

**Table 1:** Effects of MLB and ZLB on weight gain (g) in rats fed a normal diet or a high-fat diet for 4 weeks.

	Group	NC	HC	MLB 10	ZLB 1	ZLB 2.5	ZLB 5	ZLB 10
Day								
**1**		168 ± 4	166 ± 8	162 ± 8	164 ± 3	167 ± 6	153 ± 5	161 ± 6
**5**		216 ± 6	218 ± 5	213 ± 10	213 ± 6	223 ± 6	207 ± 6 ^†^	209 ± 3
**8**		242± 8	247 ± 7	238 ± 10	240 ± 9	251 ± 8	234 ± 6 ^†^	233 ± 4 ^†^
**12**		280 ± 11	299 ± 7 ^**^	284 ± 13 ^†^	287 ± 11	296 ± 11 ^*^	276 ± 9 ^†††^	274 ± 6 ^†††^
**16**		311 ± 12	345 ± 8 ^***^	320 ± 17 ^†††^	333 ± 14 ^**^	335 ± 10 ^***^	315 ± 8 ^†††^	310 ± 5 ^†††^
**21**		339 ± 19	396 ± 15 ^***^	361 ± 18 ^*,†††^	375 ± 16 ^***,†^	379 ± 16 ^***^	355 ± 8 ^†††^	352 ± 5 ^†††^
**26**		357 ± 13	421 ± 18 ^***^	380 ± 14 ^*,†††^	413 ± 22 ^***^	404 ± 17 ^***^	389 ± 8 ^**,††^	372 ± 15 ^†††^
**30**		376 ± 17	448 ± 21 ^***^	400 ± 17 ^*,†††^	441 ± 21 ^***^	425 ± 15 ^***,†^	400 ± 8 ^*,***^	395 ± 14 ^†††^

NC, normal diet control; HC, high-fat-diet control; MLB 10, high-fat diet with 10 mg/kg/day of MLB; ZLB 1, high-fat diet with 1 mg/kg/day of ZLB; ZLB 2.5, high-fat diet with 2.5 mg/kg/day of ZLB; ZLB 5, high-fat diet with 5 mg/kg/day of ZLB; ZLB10, high-fat diet with 10 mg/kg/day of ZLB. Values are expressed as mean ± SD (n = 6 per group). ^*^P < 0.05, ^**^P < 0.01, and ^***^P < 0.001 vs. NC group, ^†^ *P* < 0.05, ^††^ *P* < 0.01, and ^†††^ *P* < 0.001 vs. HC group based on one-way ANOVA with Duncan’s test.

**Table 2:** Effects of ZLB on biochemical parameters in rats fed a normal diet or a high-fat diet for 4 weeks.

	NC	HC	MLB 10	ZLB 1	ZLB 2.5	ZLB 5	ZLB 10
**AST (U/L)**	154.3 ± 17.4	233.7 ± 39.9 ^***^	164.0 ± 24.0 ^†††^	247.3 ± 49.5 ^***^	189.5 ± 23.8 ^†^	168.5 ± 13.7 ^†††^	164.3 ± 23.1 ^†††^
**ALT (U/L)**	52.2 ± 5.5	62.7 ± 5.8 ^*^	48.5 ± 7.8 ^†^	54.0 ± 7.0	52.7 ± 9.2	49.0 ± 5.4 ^†^	44.0 ± 6.9 ^††^
**BUN (mg/dL)**	15.3 ± 2.1	11.0 ± 1.8	11.3 ± 1.8	9.8 ± 1.7	11.3 ± 1.0	11.5 ± 1.0	11.8 ± 1.0
**Creatinine (mg/dL)**	0.7 ± 0.1	0.7 ± 0.1	0.7 ± 0.1	0.7 ± 0.1	0.7 ± 0.1	0.6 ± 0.0	0.7 ± 0.1
**TC (mg/dL)**	47.8 ± 9.4	65.0 ± 9.3 ^**^	54.8 ± 4.6	67.3 ± 7.3 ^***^	58.3 ± 12.0	55.0 ± 11.4	51.0 ± 4.3 ^††^
**TAG (mg/dL)**	26.5 ± 4.5	42.8 ± 8.0 ^**^	31.2 ± 3.8 ^†††^	43.5 ± 5.0 ^**^	45.2 ± 5.7 ^**^	33.3 ± 3.4	33.7 ± 7.1
**non-HDL-C (mg/dL)**	18.2 ± 7.4	28.2 ± 5.1 ^**^	19.7 ± 8.5 ^†^	20.3 ± 2.3 ^†^	17.5 ± 5.1 ^††^	18.5 ± 3.0 ^††^	18.2 ± 4.3 ^††^
**HDL (mg/dL)**	28.2 ± 6.7	16.5 ± 5.9 ^**^	28.3 ± 4.5 ^††^	24.7 ± 7.5 ^†^	26.2 ± 5.7 ^†^	28.5 ± 9.5 ^††^	25.2 ± 7.3 ^†^
**TNFα (pg/dL)**	0.72 ± 0.25	10.66 ± 2.66 ^***^	3.36 ± 1.77 ^††^	7.21 ± 1.69^**^	6.66 ± 1.44 ^**^	5.30 ± 1.3 ^*, †^	5.22 ± 0.94 ^*, †^
**Leptin (pg/dL)**	94.65 ± 4.71	252.57 ± 45.46 ^***^	148.08 ± 46.89 ^††^	199.10 ± 20.25 ^*^	204.89 ± 38.60 ^*^	151.88 ± 13.57 ^†^	155.66 ± 15.36 ^†^

AST, aspartate transaminase; ALT, alanine transaminase; BUN, blood urea nitrogen; TC, total cholesterol; TAG, triacylglycerol; HDL, high-density lipoprotein cholesterol; TNFα, tumor necrosis factor α; NC, normal diet control; HC, high-fat-diet control; MLB 10, high-fat diet with 10 mg/kg/day of MLB; ZLB 1, high-fat diet with 1 mg/kg/day of ZLB; ZLB 2.5, high-fat diet with 2.5 mg/kg/day of ZLB; ZLB 5, high-fat diet with 5 mg/kg/day of ZLB; ZLB10, high-fat diet with 10 mg/kg/day of ZLB. Values are expressed as mean ± SD (n = 6 per group). ^*^P < 0.05, ^**^P < 0.01, and ^***^P < 0.001 vs. NC group, ^†^ *P* < 0.05, ^††^ *P* < 0.01, and ^†††^ *P* < 0.001 vs. HC group based on one-way ANOVA with Duncan’s test.

**Table 3:** Effects of MLB and ZLB on the glucose tolerance the levels of blood glucose were measured every 30 min for 2 h three weeks after administration of MLB or ZLB.

	Group	NC	HC	MLB 10	ZLB 1	ZLB 2.5	ZLB 5	ZLB 10
Time (min)								
**0**		97 ± 2	115 ± 9 ^***^	104 ± 4	112 ± 5 ^***^	112 ± 6 ^***^	103 ± 5 ^††^	104 ± 3 ^††^
**30**		473 ± 55	578 ± 30 ^***^	510 ± 16 ^††^	579 ± 31 ^***^	524 ± 45 ^*,†^	506 ± 32 ^††^	509 ± 38 ^††^
**60**		295 ± 31	445 ± 70 ^***^	335 ± 38 ^††^	439 ± 44 ^***^	387 ± 90 ^*^	367 ± 41 ^†^	357 ± 41 ^†^
**90**		155 ± 10	252 ± 18 ^***^	203 ± 24 ^**,†^	273 ± 23 ^***^	210 ± 47 ^**,†^	207 ± 34 ^**,†^	206 ± 26 ^**,†^
**120**		126 ± 9	179 ± 9 ^***^	153 ± 21 ^*,†^	176 ± 13 ^***^	177 ± 30 ^***^	156 ± 20 ^*^	154 ± 13 ^*,†^

NC, normal diet control; HC, high-fat-diet control; MLB 10, high-fat diet with 10 mg/kg/day of MLB; ZLB 1, high-fat diet with 1 mg/kg/day of ZLB; ZLB 2.5, high-fat diet with 2.5 mg/kg/day of ZLB; ZLB 5, high-fat diet with 5 mg/kg/day of ZLB; ZLB10, high-fat diet with 10 mg/kg/day of ZLB. Values are expressed as mean ± SD (n = 6 per group). ^*^P < 0.05, ^**^P < 0.01, and ^***^P < 0.001 vs. NC group, ^†^ *P* < 0.05, and ^††^ *P* < 0.01 vs. HC group based on one-way ANOVA with Duncan’s test.
